# Case Report: Partial response to single-agent pembrolizumab in a chemotherapy-resistant metastatic pancreatic cancer patient with a high tumor mutation burden

**DOI:** 10.3389/fonc.2023.1118633

**Published:** 2023-03-01

**Authors:** Mengyao Dai, Jianpeng Sheng, Qi Zhang, Jianxin Wang, Qihan Fu, Tingbo Liang

**Affiliations:** ^1^ The Key Laboratory of Pancreatic Diseases of Zhejiang Province, Hangzhou, China; ^2^ The Innovation Center for the Study of Pancreatic Diseases of Zhejiang Province, Hangzhou, China; ^3^ Zhejiang Clinical Research Center of Hepatobiliary and Pancreatic Diseases, Hangzhou, China; ^4^ Department of Medical Oncology, The First Affiliated Hospital, Zhejiang University School of Medicine, Hangzhou, China; ^5^ Department of Hepatobiliary and Pancreatic Surgery, The First Affiliated Hospital, Zhejiang University School of Medicine, Hangzhou, China

**Keywords:** molecular alterations, pancreatic cancer, immunotherapy, tumor mutation burden (TMB), cyTOF

## Abstract

Single-agent immune checkpoint blockade has shown no clinical benefits in pancreatic cancer. Recently, the programmed cell death protein 1 (PD-1) antibody pembrolizumab has been recommended as a treatment option for high tumor mutational burden (TMB) solid tumors based on the data from a basket trial. However, no pancreatic cancer patients were enrolled in that trial. Whether pancreatic cancer patients with high TMB respond to PD-1 blockade as well remains unclear. Here, we report a case with a partial response to single-agent immunotherapy with pembrolizumab in pancreatic cancer with high TMB after the failure of several lines of chemotherapy. This result indicates that single-agent immunotherapy may be effective in pancreatic cancer patients with high TMB. In addition, in order to understand the basic immune state of our patients, we also analyzed the changes in immune cells in peripheral blood with cytometry by time-of-flight mass spectrometry (CyTOF) before and after pembrolizumab treatment.

## 1 Introduction

Pancreatic cancer is one of the leading causes of cancer-related death (1). Patients with metastatic disease had grim prognoses; despite intensive chemotherapy, their overall survival was only 8.5–11.1 months (2, 3). More novel systematic treatment options are urgently needed.

Immune checkpoint blockade (ICB) therapy is showing promising results in a variety of solid tumors (4, 5). However, because few patients respond to single-agent immunotherapy, it is not recommended for pancreatic cancer (6, 7). One strategy for increasing efficacy is to identify a subset of patients who may benefit from immunotherapy using biomarkers.

Microsatellite instability - high (MSI-H), programmed cell death ligand 1 (PD-L1) expression, and high tumor mutational burden (TMB) are the most actively investigated biomarkers (8). Other factors with antitumor activity (e.g., microRNAs, B-cell lymphoma-2 (Bcl-2), and C-kit) were also investigated in preclinical studies; however, those factors were not found to be good predictors of the effect of immunotherapy at this time (9–12).

MSI-H has been established as a good predictor of response. In the KEYNOTE-158 trial, 233 patients with MSI-H tumors were treated with pembrolizumab, and the objective response rate (ORR) was 34.3%. In this trial, 22 pancreatic cancer patients with MSI-H were included, and the ORR was 18.2%. Consistent with this finding, all six pancreatic cancer patients with MSI-H tumors enrolled in the basket trial of Know Your Tumor responded to the programmed cell death protein 1 (PD-1) antibody treatment (13). The duration of response was relatively long, with four cases still on treatment for more than 10 months at the data cutoff. Based on these data, pembrolizumab is approved for the treatment of solid tumors with MSI-H and was also recently recommended as a treatment option for pancreatic cancer in the NCCN guidelines. PD-L1 expression demonstrated some success in identifying patients most likely to benefit from immunotherapy in KEYNOTE-028. This cohort included 24 pancreatic cancer patients with PD-L1-positive expression; however, their ORR was 0%. The findings showed that PD-L1 expression does not predict pancreatic cancer response.

In the biomarker analysis parts of KEYNOTE-158, 102 patients with TMB of ≥ 10 mutations (mut)/Mb were evaluated; four (3.9%) had a complete response (CR) and 26 (25.5%) had a partial response (PR); the ORR was 29.4%. In contrast, a lower ORR of 6.3% was observed in patients with TMB of < 10 mut/Mb (14). Based on the results of this trial, the FDA approved pembrolizumab for the treatment of TMB-H advanced solid tumors after the failure of previous routine chemotherapy. However, this trial supporting the use of pembrolizumab in TMB-H solid tumors did not include patients with pancreatic cancer. Other studies exploring the correlation between TMB and the efficacy of immunotherapy did not include patients with pancreatic cancer either. There is only one case report describing a patient with pancreatic cancer who achieved PR with the combination treatment of pembrolizumab and the antiangiogenic drug lenvatinib (15). As a result, it is unknown whether patients with pancreatic cancer with high TMB can benefit from pembrolizumab.

Herein, we present the first case, to our knowledge, of a partial response to a single-agent anti-PD-1 antibody in a patient with advanced pancreatic cancer and a high level of TMB, and we investigate the change in the underlying immune state.

## 2 Case presentation

A 55-year-old man presented with abdominal pain in the fall of 2019. On 14 August, he underwent an endoscopic ultrasound-guided fine-needle aspiration biopsy (EUS-FNA) and was diagnosed with pancreatic cancer, demonstrating a poorly differentiated adenocarcinoma. His clinical stage was T1N2M1. His CA199 was negative, while his CEA and AFP were abnormal. He received first-line chemotherapy with gemcitabine and Abraxane for six cycles; he partially responded to this regimen and had a progress-free survival (PFS) of 4 months. Afterward, he underwent eight doses of chemotherapy with modified oxaliplatin, irinotecan, and 5-fluorouracil (FOLFRINOX). The effect was also evaluated as PR, and the PFS was 5.5 months. He tried raltitrexed; subsequently, the lesion grew slowly, and the efficacy was stable disease (SD).

**Figure 1:**
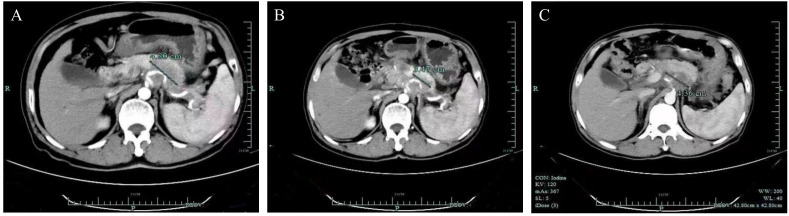
Computed tomography scans of the targeted lesion. **(A)** Baseline. **(B)** After three doses of pembrolizumab, demonstrating a partial response (PR) of the lesion in the pancreas. **(C)** After six doses of treatment, the lesion was enlarged.

He did genetic testing to see if there was an option for targeted therapy. Given that his biopsy specimen was not enough for the next-generation-sequencing (NGS) testing, molecular profiling was performed with circulating tumor DNA (ctDNA) on 3 July 2020. The results demonstrated that the tumor mutation burden (TMB) was 68.74 mut/MB. In addition, many molecular alterations were found (**Table 1**), with the TSC2 p.V1069Dfs*98 frameshift mutation in exon 28 potentially actionable with a drug available in China. Given the high TMB levels in his blood and the lack of standard treatment options, the decision was made to start pembrolizumab at 100 mg every 3 weeks after discussing the rationale and potential risks and benefits. He was in good condition before the treatment and tolerated the immunotherapy very well, with no adverse events observed. His cancer-related pain was significantly reduced 1 week after the first dose, with a numerical rating scale (NRS) decreasing from 4 to 0. After three doses, a CT scan was performed on 28 August 2020 and revealed a partial response of the pancreatic lesion (**Figure 1**). However, a subsequent CT scan on 29 October 2020 revealed that his disease had progressed. The patient had received a total of six cycles of pembrolizumab, with a PFS of 4.3 months.

**Table 1:** Summary of molecular pathology.

Molecular analysis (ctNDA NGS)	
TMB 68.74 mt/MB	Microsatellite state, CPS, and TPS not applicable
*TSC2* p.V1069Dfs*98 frameshift mutation in exon 28: 19.8%	*ARID1A* p.Q758Rfs*75 frameshift mutation in exon 7: 40.3%
*ARID2* p.E533 frameshift mutation in exon 13: 13.4%	*AXIN2* p.N666Qfs*41 frameshift mutation in exon 8: 19.3%
*BAK1* p.F35Sfs*113 frameshift mutation in exon 3: 17.3%	*BAX* p.R89Efs*44 frameshift mutation in exon 4: 18.5%
*BAX* p.E41Rfs*19 frameshift mutation in exon 3: 16.1%	*BRAF* p.D594G missense mutation in exon 15: 6.6%
*CDH1* pG169Afs*46 frameshift mutation in exon 4: 16.5%	*CDH1* p.P126Rfs*89 frameshift mutation in exon 3: 14.6%
*CYP2D6* p.R218Pfs*4 frameshift mutation in exon 4: 31.5%	*FLCN* p.H429Pfs*27 frameshift mutation in exon 11: 19.3%
*GNAS* p.R201C missense mutation in exon 8: 20.0%	*JAK2* p.W157* truncation mutation in exon 6: 2.1%
*KMT2B* p.A1049Gfs*39 frameshift mutation in exon 8: 27.0%	*LRP18* p.L4076Wfs*31 frameshift mutation in exon 80: 17.6%
*MLH1* p.K196Nfs*6 frameshift mutation in exon 7: 16.6%	*ARID1A* p.H2018Y missense mutation in exon 20: 46.2%
*ARID1A* p.I847T missense mutation in exon 8: 14.4%	*ATR* p.L618del non-shift deletion mutation in exon 8: 15.5%
*BAK1* p.Q73K missense mutation in exon 4: 17.5%	*BRCA1* p.L1729M missense mutation in exon 19: 2.0%
*BRIP1* p.A1030V missense mutation in exon 20: 2.8%	*CDH1* p.E165D missense mutation in exon 4: 19.8%
*CDKN1B* p.*199Qext*60 read through mutation in exon 2: 19.8%	*CDKN2A* p.R107C missense mutation in exon 2: 3.7%
*DDR2* p.R135C missense mutation in exon 6: 0.9%	*DLL3* p.L375M missense mutation in exon 7: 19.2%
*DNMT3A* p.A84D missense mutation in exon 4: 0.9%	*EPAS1* p.L432M missense mutation in exon 10: 2.4%
*EPHA2* p.R762H missense mutation in exon 13: 14%	*FANCC* p.A421T missense mutation in exon 13: 15.3%
*FAT1* p.R2021H missense mutation in exon 10: 18.2%	*GATA6* p.S364N missense mutation in exon 2: 14.9%
*GRM3* p.E573D missense mutation in exon 4: 3.5%	*GRM3* p.N251I missense mutation in exon 23: 2.4%
*KDM5A* p.R1167 missense mutation in exon 23: 17.3%	*LHCGR* p.R43H missense mutation in exon 1: 17.0%
*MAP2K1* p.F53L missense mutation in exon 2: 1.2%	*MLLT1* p.D103Y missense mutation in exon 4: 20.8%
*MTOR* p.M1038V missense mutation in exon 20: 2.3%	*NKX2-1* p.A299T missense mutation in exon 2: 1.6%
*NOTCH2* p.A1403T missense mutation in exon 25: 8.8%	*PTCH1* p.R294H missense mutation in exon 6: 2.1%
*RAC3* p.C105R missense mutation in exon 5: 1.0%	*RAD51D* p.R291H missense mutation in exon 9: 21.0%
*RECQL4* p.R849C missense mutation in exon 15: 2.1%	*SRC* p.A197T missense mutation in exon 8: 16.1%
*SUFU* p.A405V missense mutation in exon 10: 15.3%	*UGT1A1* p.R523Efs*22 frameshift mutation in exon 5: 8.8%

Following that, the patient was given capecitabine. On 26 May 2021, he was admitted to the emergency room because of abdominal pain and bloody stools. He was diagnosed with massive gastrointestinal bleeding combined with hemorrhagic shock and eventually died. His overall survival was 21.7 months.

**Figure 2:**
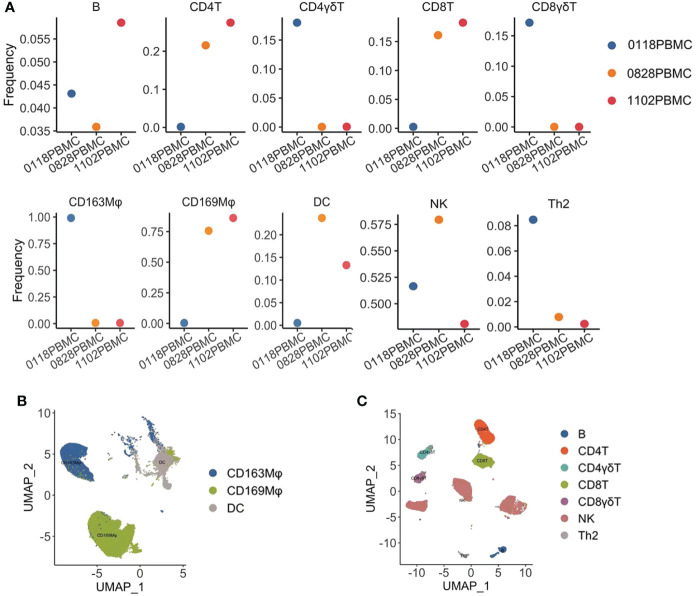
Cytometry by time-of-flight mass spectrometry (CyTOF) analysis of the immune state of the patient. **(A)** Frequencies of immune cell subsets in the peripheral blood mononuclear cell (PBMC) of a patient during treatment with pembrolizumab, 0118: before treatment, 0828: when the patient achieved PR, and 1102: when the patient’s disease had progressed. The frequencies of B cells, CD4^+^T cells, CD8^+^T cells, CD4^+^γδT cells, CD8^+^γδT cells, CD163^+^ macrophages, CD169^+^ macrophages, dendritic cells (DCs), natural killer cells (NKs), and T helper 2 cells (Th2) were analyzed. **(B)** Uniform Manifold Approximation and Projection (UMAP) displayed the distribution of myeloid cells from the PBMC in the patient. **(C)** UMAP displayed the distribution of lymphocytes from the PBMC in the patient.

In order to know further about how the immune state changed in a pancreatic cancer patient who responded to monotherapy with pembrolizumab, we analyzed the immune cells in peripheral blood by cytometry by time-of-flight mass spectrometry (CyTOF) (**Figure 2**). We collected the blood sample at different stages of treatment (the first stage: before treatment, the second stage: when the patient was in partial remission after 2 months of treatment, and the third stage: when the patient progressed after 4 months of treatment). Before treatment, the frequencies of CD4^+^T cells, CD8^+^T cells, CD4^+^γδT cells, CD8^+^γδT cells, natural killer cells (NKs), and dendritic cells (DCs) were low, indicating that the patient was in an immunosuppressed state with a decreased immune cell number. Compared with baseline, the frequency of the CD4^+^T cells and CD8^+^T cells was significantly increased after treatment, and the frequency of NKs and DCs was slightly increased, suggesting that for this patient, immunotherapy was effective and his immune state was restored. In contrast, the frequency of CD4^+^γδT cells and CD8^+^γδT cells was reduced even further. Interestingly, we have found some changes in the macrophage subset. The frequency of CD169^+^ macrophages was low before the treatment, but it went up when the patient had a partial response to pembrolizumab. In contrast, the frequency of CD163^+^ macrophages was high at baseline and decreased to an extremely low level during the treatment. The proportion of B cells and Th2 cells was slightly decreased, which may indicate that the patient’s humoral immune function was low.

The investigators obtained informed consent to publish information and images from the patients.

## 3 Discussion

Pancreatic cancer is one of the most deadly cancers (16). Its poor prognosis is associated with difficulty in early diagnosis, high metastatic potential, and a poor response to chemoradiotherapy. Given the distinct nature of tumor cells and their surrounding matrix, pancreatic ductal adenocarcinoma (PDAC) appears to exhibit immune evasion early on, with poor response to immune checkpoint inhibitors such as PD-1 or anticytotoxic T lymphocyte-associated antigen 4 (CTLA-4) (17).

This case report shows a male patient with a high level of TMB who responded to pembrolizumab after previous treatment with GA, modified FOLFRINOX, and raltitrexed. TMB-H is a rare event in PDAC, accounting for approximately 1.1% of cases (18). Even in TMB-H PDAC patients, the mean TMB was only 37.6 mut/Mb (18). Our patient has a TMB of 68 mut/Mb, which is considerably high, and he responds to immunotherapy. However, his PFS lasts only for 2 months, which is unsatisfactory. Matched therapy based on molecular profiling is now routine care to guide the treatment of patients with advanced solid tumors, and the adoption of precision medicine can also have a substantial effect on patients with pancreatic cancer (13, 19). Most pancreatic cancers have somatic mutations (20). The most common genetic changes in PDAC are oncogenic KRAS mutations and tumor suppressor inactivation of CDKN2A, TP53, and SMAD4 (13). Several genetic mutations were found in this case, among which TSC2 alternation is considered actionable. The product of the *TSC2* gene is considered to be a tumor suppressor. A TSC2 mutation may lead to a disorder of the PTEN/PI3K/Akt pathway and overactivation of mTORC1, while a disorder of the mTOR signal can promote excessive tumor growth, which is common in some tumors (21). Somatic mutation in TSC2 predicts response to mTOR inhibitors in renal cell carcinoma (22). Therefore, an mTOR inhibitor may be a potential treatment for tumors with TSC2 mutations. In addition, a tumor with a TSC2 alteration is potentially responsive to immunotherapy. A case report showed that a combination of chemotherapy and sintilimab effectively treated metastatic pancreatic cancer patients with SMAD4 and TSC2 mutations after the failure of prior chemotherapy (23). Preclinical studies show that treatment of immunotherapy can inhibit the growth of TSC2-deficient tumors and enhance T-cell infiltration (24). Therefore, the combination of mTOR inhibitors and PD-1 inhibitors may be a potential therapeutic strategy for pancreatic cancer with high TMB levels and a TSC2 mutation. This patient did not try the combination treatment of a PD-1 inhibitor and a mTOR inhibitor, which may further increase the therapeutic effect.

In the advanced stage of pancreatic cancer, tumor cells widely inhibit immune function, resulting in an imbalance of immune cell subsets. Therefore, effective immunotherapy is of great significance for improving the survival and symptoms of patients. In this case, we analyzed the changes in immune cell subsets in patients with pancreatic cancer before and after treatment with PD-1 inhibitors to learn more about their immune state.

CD4 is mainly expressed in helper T cells (Th cells), and CD8 is mainly expressed on the surface of suppressor T cells (Ts cell) and cytotoxic T cells (Tc cell). Circulating CD4^+^T cells and CD8^+^T cells are associated with a good prognosis in patients (25). In this case, before immunotherapy, the frequency of CD4^+^ and CD8^+^T cells in the peripheral blood of patients was extremely low, suggesting a great defect in immune function. After treatment, the frequency of CD4^+^ and CD8^+^T cells was significantly increased, indicating that the immune state of the patient was constantly recovering. Consistent with previous studies, our findings suggested that PD-1 blockade has a regulatory effect on the immune state (26, 27).

The majority of mature T lymphocytes in peripheral blood were αβ T cells, and a few were γδT cells (28). We found that the frequency of CD4^+^γδT cells and CD8^+^γδT cells was relatively high at baseline and decreased during treatment. It is well known that γδT cells play an important role in the host’s natural defense against infection and malignant tumors. γδT-cell infiltration is associated with a good prognosis (28, 29). Other studies have shown that PD-1 checkpoint blockade combined with γδT-cell immunotherapy could effectively inhibit the growth of prostate tumor cells (30). Therefore, PD-1/PD-L1 inhibitors combined with γδT-cell immunotherapy may be beneficial for this patient.

We have found an increase in CD169^+^ macrophage and a decrease in CD163^+^ macrophage subsets. CD169^+^ macrophages mainly play a role in inhibiting cancer (31). CD163 can be used as a specific marker of M2-type macrophages (32). The existence of CD163^+^ macrophages has been detected in multiple types of solid tumors, and the infiltration degree of CD163^+^ macrophages is related to early recurrence and reduced survival (33). M2 macrophage subsets could promote T-cell apoptosis through the PD-L1/PD-1 pathway (34). Depletion of CD163^+^ TAM facilitated the recruitment of CD4^+^ and CD8^+^T cells (35). The reduced frequency of CD163^+^ macrophages during pembrolizumab treatment in this patient could be the mechanism of the antitumor effect. It is not clear how pembrolizumab causes the decrease of CD163^+^ macrophage frequency. It is worth further exploring whether and how those unique macrophages were involved in the antitumor immunity of pembrolizumab.

DCs could dictate the responses of immunotherapy. PD-L1 blockade can activate DCs to enhance T-cell initiation and improve its antitumor effect (36). We found that the proportion of DCs peaked during pembrolizumab treatment and decreased gradually at the end of the treatment. Blocking of PD-1 may activate the antitumor effects of DCs.

Taken together, we have found that this patient with a high level of TMB responded to pembrolizumab, and several types of immune cells were changed accordingly during the treatment. Limited by the fact that we of lacked a biopsy specimen, we only analyzed a blood sample in this case; how the immune state changed in the tumor environment was not known. Further studies are needed to explore the characteristics and functions of immune cells in pancreatic cancer patients who respond to pembrolizumab and actively look for potential predictive markers.

## 4 Conclusions

In this case, a pancreatic cancer patient with a high level of TMB and TSC2 mutations responds to single-agent immunotherapy. Analysis of circulating immune cells has also provided multiple insights into the different stages of treatment, deepening the understanding of immune cell function in pancreatic cancer.

## Data availability statement

The raw data supporting the conclusions of this article will be made available by the authors, without undue reservation.

## Ethics statement

The studies involving human participants were reviewed and approved by Clinical Research Ethics Committee, The First Affiliated Hospital of Zhejiang University School of Medicine. The patients/participants provided their written informed consent to participate in this study. Written informed consent was obtained from the individual(s) for the publication of any potentially identifiable images or data included in this article.

## Author contributions

Conception and design: LT and QF. Provision of study material or patients: QF. Collection and assembly of data: MD. Data analysis and interpretation: MD. Manuscript writing: MD and QF. All authors contributed to the article and approved the submitted version.
